# Prognostic Significance of Serum Inflammatory Markers in Gastric Cancer

**DOI:** 10.1007/s11605-017-3597-5

**Published:** 2017-10-04

**Authors:** Arfon G. M. T. Powell, Debora Parkinson, Neil Patel, David Chan, Adam Christian, Wyn G. Lewis

**Affiliations:** 1 Division of Cancer Genetics, University Hospital of Wales, Cardiff University, Heath Park, Cardiff, UK; 2 Department of Surgery, Cardiff & Vale University Health Board, University Hospital of Wales, Heath Park, Cardiff, UK; 3 Department of Pathology, Cardiff & Vale University Health Board, University Hospital of Wales, Heath Park, Cardiff, UK

**Keywords:** Gastric cancer, Systemic inflammation, Survival

## Abstract

**Background:**

The aim of this study was to assess the relative prognostic value of biomarkers to measure the systemic inflammatory response (SIR) and improve prognostic modeling in a cohort of patients undergoing potentially curative surgery for gastric adenocarcinoma. The hypothesis was that a single SIR biomarker would be associated with the most prognostic value.

**Methods:**

Consecutive 331 patients undergoing surgery for gastric cancer between 2004 and 2016 within a regional UK cancer network were identified. Serum measurements of hemoglobin, C-reactive protein, albumin, modified Glasgow Prognostic Score, and differential white cell counts were obtained before surgery, and correlated with histopathological factors (pTNM stage, differentiation, and vascular invasion) and survival. Primary outcome measures were disease-free (DFS) and overall survival (OS).

**Results:**

Consecutive 331 patients were identified and 291 underwent potentially curative gastrectomy for adenocarcinoma. On univariable DFS analysis, female gender (*p* = 0.027), proximal location (*p* = 0.018), pT stage (*p* < 0.001), pN stage (*p* < 0.001), pTNM stage (*p* < 0.001), vascular invasion (*p* < 0.001), poor differentiation (*p* = 0.001), lymph node ratio (*p* < 0.001), R1 status (*p* < 0.001), platelet count (*p* = 0.038), and mGPS (*p* = 0.001) were significantly associated with poor survival. The mGPS was associated with advanced pT stage (*p* = 0.001), pTNM stage (*p* = 0.013), and poor differentiation (*p* = 0.030). On multivariable DFS analysis, mGPS [hazard ratio (HR) 2.51, 95% confidence interval (CI) 1.35–4.65, *p* = 0.011] was the only inflammatory marker to retain independent significance. Multivariable OS analysis revealed similar findings; mGPS (HR 2.75, (95% CI 1.65–4.59), *p* < 0.001).

**Conclusion:**

mGPS is an important and only SIR-related prognostic biomarker independently associated with both DFS and OS in gastric cancer.

**Electronic supplementary material:**

The online version of this article (10.1007/s11605-017-3597-5) contains supplementary material, which is available to authorized users.

## Introduction

The term biomarker originated in the 1950s and has been defined by the National Institute of Health as a characteristic that is objectively measured and evaluated as an indicator of normal biological processes, pathological processes, or pharmacological responses to a therapeutic intervention.^1^ Examples include everything from pulse and blood pressure, through basic biochemistry, to more complex laboratory tests of blood and other tissues. Globally, gastric cancer is the third leading cause of cancer-related death, accounting for some 740,000 deaths annually.^2^ Surgery remains the only potentially curative treatment, yet some 40% of patients develop recurrence, and the use of chemotherapy has no firmly established standard of care. Any reasonable observer therefore would recognize that a prime challenge is to identify biomarkers that may improve prognostic modeling, independent of contemporary staging, which may promote new therapeutic targets.

Cancer-related inflammation has been termed the 7th hallmark of cancer,^3^ and the systemic inflammatory response (SIR) measured using cellular (whole white cell counts, neutrophils, lymphocytes, and platelets) and humoral [C-reactive protein (CRP) and albumin] components. Derivative biomarkers (neutrophil-lymphocyte ratio (NLR),^4^ platelet-lymphocyte ratio (PLR),^5^ neutrophil-platelet score (NPS),^6^ and the modified Glasgow prognostic score (mGPS)^7^,^8^ have also been described and reported to be associated with poor survival in patients undergoing potentially curative surgery. If the SIR is to be considered a possible therapeutic target, then a single marker is desirable, that is sensitive, specific, robust, accurate, and reproducible. Reports regarding biomarker significance, in minimizing confounding variables, have only included common clinicopathological factors in multivariable regression models,^4^,^6^,^9^ and no study has examined rigorously the relative prognostic significance of the SIR’s individual components and derivative markers.

The aim of this study was to determine if a single biomarker of the SIR was independently associated with survival, in patients undergoing potentially curative gastrectomy for cancer. The hypothesis was that a composite biomarker of the SIR would have significant optimum prognostic value, independent of histopathological TNM stage, and other SIR markers on multivariable regression modeling. The setting was a regional UK cancer network serving a population of 1.8 million.

## Methods

### Patients

In order to test the hypotheses proposed in this study, a single cohort was developed and included patients with radiological TNM stage I to III, who following staging were deemed to have potentially resectable gastric cancer between January 2004 and December 2016. All patients were managed by a multidisciplinary team of specialists with an interest in gastric cancer and included surgeons, oncologists, radiologists, anesthetists, and pathologists. Preoperative staging involved computed tomography (CT) of the thorax, abdomen, and pelvis as well as a staging laparoscopy of the peritoneal cavity when appropriate, in order to facilitate individually patient tailored management plans. Patients who underwent endoscopic resection and Siewert II cancers that were managed as oesophageal cancers were not included in the study.

Selective use of neoadjuvant chemotherapy was adopted following publication of the Medical Research Council Adjuvant Gastric Infusional Chemotherapy (MAGIC) trial 10 in the latter part of the study and was prescribed to 45 patients with minimal comorbidities who were deemed to have relatively advanced disease and would benefit from down-staging of the tumor prior to surgery. Chemotherapy was administered for 3 or 4 cycles preoperatively and postoperatively. Each cycle consisted of epirubicin (50 mg/m^2^) by intravenous bolus, cisplatin (60 mg/m^2^) as a 4-h infusion on day one and 5-fluorouracil (200 mg/m^2^/day) daily by a continuous intravenous infusion.

The type of surgery for gastric cancer was determined by the anatomical location of the tumor; subtotal gastrectomy was performed in patients with antral tumors and total gastrectomy was performed in patients with tumors of the cardia (Siewert type III), body, and linitis plastic. A modified extended D2 lymphadenectomy (preserving pancreas and spleen where possible) was performed and the operative approach was open in all cases.

Ethical approval was sought, but the chair of Cardiff & Value University Health Board ethics committee confirmed that individual patient consent was not required to report clinical outcomes alone, and no formal approval was necessary.

### Clinicopathological Characteristics

Tumors were staged using the seventh edition of the AJCC/UICC-TNM staging system. Pathological factors were recorded from pathology reports issued at the time of surgery and included tumor differentiation, vascular invasion, margin status, and the number of lymph nodes with and without metastasis. The metastatic to normal lymph node ratio (LNR) was calculated by dividing the number of lymph nodes containing metastatic carcinoma to the total number of lymph nodes identified. The LNR was then categorized as low risk < 0.25 and high risk > 0.50 within the TNM stage III.^11^

Routine laboratory measurements of whole white cell count, neutrophil count, lymphocyte count, platelet counts, CRP, and albumin were taken on the day prior to surgery. Derivate measurements of systemic inflammation consisted of the NLR, NPS, PLR, and the mGPS were calculated. The NLR and PLR were constructed by calculated the neutrophil to lymphocyte ratio and the platelet to lymphocyte ratio, respectively. These derivative measurements were then dichotomized into low and high groups by 5.5 for NLR and 150 for PLR.^11^ The NPS was constructed by grouping patients into three groups; 0 for patients with both normal neutrophil (≤ 7.5 × 10^9^/L) and platelet counts (≤ 400 × 10^9^/L), 1 for patients with either a high neutrophil (> 7.5 × 10^9^/L) or platelet count (> 400 × 10^9^/L), and 2 for patients with both high neutrophil and platelet counts. The mGPS was constructed using CRP and albumin. Patients with normal serum levels of CRP (≤ 10 mg/L) and albumin (≥ 35 g/L) were given a score of 0. Patients with a raised serum CRP (> 10 mg/L) and normal serum albumin were given a score of 1 and patients with a raised serum CRP and low serum albumin (< 35 g/L) were given a score of 2.^12^

Patients were followed up at regular intervals of 3 months for the first year and 6 months thereafter. In the event that patients developed symptoms suggestive of recurrent disease, investigations were undertaken sooner. Follow-up surveillance was conducted for 5 years or until death whichever was sooner. Overall survival was calculated from time of diagnosis to the date of death. Disease-free survival was measured from the date of surgery until the date of recurrence or date of censoring. The median follow-up was 60 months (range 6 to 60), with 255 patients (77%) followed up for 5 years or until death. No patients were lost to follow-up. Death certification was obtained from the Office for National Statistics via Cancer Network Information System Cymru (CaNISC). Patterns of recurrence were defined as locoregional, distant (metastatic), or both locoregional and distant, when both were diagnosed at the same time. The time of recurrence was taken as the date of the confirmatory investigation.

### Statistical Analysis

#### Justification of Sample Size

Sample size calculations were based on a prestudy literature survey of (CRUK cancers statistics),^13^ which indicated that the baseline 5-year survival rate of patients diagnosed with stage I gastric cancer was expected to be 80%, compared with 60% in patients with stage II gastric cancer, and a 15% difference in survival would be a realistic expectation. Thus, a minimum of 276 patients were to be studied, providing 80% power to detect such a difference with *p* < 0.05.

#### Methods of Data Analysis

Grouped data were expressed as median (range) and nonparametric methods used throughout. Patient demographics were analyzed between the treatment modalities by means of *χ*2 or nonparametric tests, including Mann-Whitney *U* test. These tests were also employed in the analysis of disease recurrence and time to recurrence for the treatment groups. Disease-free survival for all patients was calculated by measuring the interval from a landmark time of 6 months after diagnosis to the date of recurrence. This approach was adopted in previous randomized trials,^10^–^14^ to allow for the variable interval to surgery following diagnosis, depending on whether neoadjuvant therapy was prescribed. As in these trials, events resulting in a failure to complete curative treatment, such as not proceeding to surgery, open and close laparotomy, palliative resection, in-hospital mortality, and disease progression during neoadjuvant chemotherapy, were assumed to have occurred at this landmark time, to maintain the intention-to-treat analysis. Overall survival was measured from the date of diagnosis. Cumulative survival was calculated according to the method of Kaplan and Meier; differences between groups were analyzed with the log-rank test. Univariable analyses examining factors influencing survival were examined initially by the life table method of Kaplan and Meier, and those with associations found to be significant (*p* < 0.010) were retained in a Cox proportional hazards model using forward conditional methodology to assess the prognostic value of individual variables. All statistical analysis was performed in SPSS^®^ (IBM^®^ SPSS^®^ Statistics v23.0.0.0, IBM Corporation, Armonk, New York, USA) with extension R.

## Results

### Patients, Clinicopathological Factors, and Features Associated with Nonresectability

In total, 331 patients were identified who underwent surgery for gastric cancer. Unfortunately, 41 (12.3%) patients were deemed not to have resectable tumors because of local invasion and those at risk of obstruction underwent a palliative bypass. The remainder, 291 patients, underwent potentially curative modified D2 gastrectomy. The patient cohort undergoing palliative surgery had a higher proportion of males (*p* = 0.002), raised serum CRP measurements (*p* = 0.003), hypoalbuminaemia (*p* = 0.024), higher mGPS (*p* = 0.001), and a fewer patients had undergone neoadjuvant chemotherapy (*p* = 0.024, supplementary table 1). On multivariable binary logistical regression analysis of significantly associated factors on univariable analysis, only male gender (odds ratio (OR) 4.66 (95% confidence interval (95% CI 1.60–13.58) *p* = 0.005), and the mGPS (OR 1.92 (1.27–2.88) *p* = 0.002) were independently associated with nonresectability. The area under the curve (AUC) for male gender was 0.62 (95% CI 0.54–0.70, *p* = 0.015) and the AUC for mGPS was 0.61 (95% CI 0.51–0.71, *p* = 0.021).

### Resection Cohort Demographics

The complete baseline characteristics of all clinicopathological variables studied can be found in Tables 1 and 2. The median age for patients undergoing resection was 69 years (inter-quartile range (IQR) 55–83) with the majority (42.1%) being between 65 and 75 years of age (Table 2). The majority of patients were male (66.7%), had distal cancer (45.0%), and were lymph node positive (55.0%). Neoadjuvant chemotherapy was prescribed to 45 patients (15.5%), and 61 patients (21%) received postoperative adjuvant chemotherapy (Table 1). During follow-up, 81 patients (27.8%) developed cancer recurrence and 109 patients (37.5%) died. Median follow-up of survivors was 60 (range 6–60) months.

**Table 1:** The relationship between tumor-related factors, overall survival, and disease-free survival in patients undergoing potentially curative resection for gastric cancer

Clinicopathological variables	Frequency *n* (%)	Disease-free survival	*p* value	Overall survival	*p* value
		5-year survival rate (%)		5-year survival rate (%)	
Tumor site					
Proximal	95 (32.6)	36.1	0.001	39.3	0.002
Body	65 (22.3)	65.9		65.9	
Distal	131 (45.0)	62.6		65.9	
< 15	157 (54.0)	56.0		57.8	
T stage					
1	66 (23.0)	89.6	< 0.001	89.6	< 0.001
2	27 (9.3)	78.6		85.7	
3	105 (36.1)	49.3		52.2	
4	92 (31.6)	30.8		33.8	
N stage					
0	131 (45.0)	73.7	< 0.001	75.8	< 0.001
1	63 (21.6)	55.6		58.3	
2	52 (17.9)	27.3		30.3	
3	45 (15.5)	21.4		25.0	
Tumor stage					
I	79 (27.1)	90.4	< 0.001	90.4	< 0.001
II	92 (31.6)	59.4		65.2	
III	120 (41.2)	25.0		26.4	
Differentiation					
Well/moderate	150 (51.5)	62.3	0.029	65.1	0.022
Poor	141 (48.5)	46.7		48.9	
Vascular invasion					
No	168 (57.2)	65.9	< 0.001	68.2	< 0.001
Yes	123 (42.3)	32.8		35.9	
Lymph node ratio					
0	131 (45.0)	73.7	< 0.001	75.8	< 0.001
0.01–0.24	75 (25.8)	53.2		55.3	
0.25–0.49	54 (18.6)	20.0		23.3	
≥ 0.50	31 (10.7)	20.0		25.0	
Lymph node sample					
≥ 15	134 (46.0)	54.0	0.786	57.5	0.963
R status					
0	246 (84.5)	61.0	< 0.001	64.0	< 0.001
1	45 (15.5)	12.5		12.5	
Adjuvant chemotherapy					
No	230 (79.0)	69.9%	0.725	60.8%	0.147
Yes	61 (21.0)	66.7%		46.7%	
Neoadjuvant therapy					
No	246 (84.5)	56.3	0.338	59.1	0.227
Yes	45 (15.5)	45.0		45.0

**Table 2:** The relationship between patient-related factors, overall survival, and disease-free survival in patients undergoing potentially curative resection for gastric cancer

Clinicopathological variables	Frequency *n* (%)	Disease-free survival	*p* value	Overall survival	*p* value
		5-year survival rate (%)		5-year survival rate (%)	
Age (years)					
< 65	99 (34.0)	62.1	0.053	51.5	0.714
65–75	121 (41.6)	67.5		45.8	
> 75 years	71 (24.4)	83.0		44.7	
Sex					
Female	97 (33.3)	61.5	0.093	38.5	0.076
Male	194 (66.7)	73.3		51.9	
White cell count					
Low	205 (70.4)	68.6	0.938	46.7	0.823
Normal	63 (21.6)	71.1		51.1	
High	23 (7.9)	71.4		42.9	
Neutrophil count					
Low	264 (90.7)	68.7	0.507	46.4	0.326
High	27 (9.3)	76.5		58.8	
Lymphocyte count					
Low	27 (9.3)	70.6	0.908	29.4	0.236
Normal	250 (85.9)	69.6		49.7	
High	14 (4.8)	62.5		37.5	
Platelet count					
Low	260 (89.3)	71.3	0.109	49.6	0.119
High	31 (10.7)	54.5		31.8	
C-reactive protein					
Normal	231 (79.4)	73.4	0.020	51.9	0.016
High	60 (20.6)	54.8		31.0	
Albumin					
Normal	219 (75.3)	70.1	0.680	49.7	0.209
Low	72 (24.7)	66.7		38.5	
Derivative markers					
Neutrophil-lymphocyte					
Ratio					
Low	261 (89.7)	70.7	0.266	47.1	0.800
High	30 (10.3)	59.1		50.0	
Neutrophil-platelet score					
Low	239 (82.1)	71.4	0.075	49.7	0.011
Intermediate	46 (15.8)	54.8		29.0	
High	6 (2.1)	100.0		100.0	
Platelet-lymphocyte					
Ratio					
Low	143 (49.1)	73.5	0.215	54.1	0.063
High	148 (50.9)	65.3		40.8	
Modified Glasgow Prognostic Score					
Low	231 (79.4)	73.4	0.044	51.9	0.015
Intermediate	31 (10.7)	52.2		34.8	
High	29 (10.0)	57.9		26.3	

### Relationships Between Clinicopathological Factors and Disease-free Survival

**Fig. 1:**
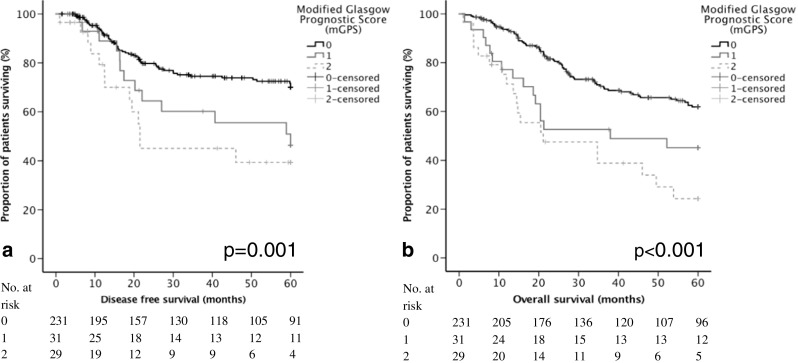
The relationship between mGPS and survival; disease-free **a** and overall survival **b**

The relationship between disease-free survival and all studied factors can be found in supplementary table 2. On univariable analysis, female gender, proximal location, advancing T stage, advancing N stage, advancing TNM stage, poor differentiation, vascular invasion, advancing lymph node ratio, R1 status, higher platelet count, raised CRP, and advancing mGPS (Fig. 1) were all associated with poor disease-free survival (*p* < 0.005) (Table 3). On multivariable analysis, only TNM stage (when compared with stage I, stage II hazard ratio (HR) 9.08 (2.13–38.67) *p* < 0.001 and stage III HR 15.83 (3.73–67.11) *p* < 0.001), vascular invasion (HR 1.70 (1.07–2.70) *p* = 0.024), R1 status (HR 2.50 (1.51–14.14) *p* = 0.002), and advancing mGPS (when compared with mGPS = 0, mGPS = 2 (HR 2.51 (1.35–4.65) *p* = 0.020) were independently associated with poor disease-free survival (Table 3). The 5-year disease-free survival was 51.9% for patients with a mGPS = 0, 34.8% for patients with a mGPS = 1, and 26.3% for patients with a mGPS = 2 (Table 2).

**Table 3:** Univariable and multivariable analysis of clinicopathological factors and serum inflammatory markers; disease-free and overall survival

	Univariable	*p* value	Multivariable	*p* value	Univariable	*p* value	Multivariable	*p* value
	Disease-free survival				Overall survival			
	Hazard ratio (95% CI)		Hazard ratio (95% CI)		Hazard ratio (95% CI)		Hazard ratio (95% CI)	
Sex								
Female	1	0.027		0.120				
Male	0.61 (0.39–0.95)							
Tumor site								
Proximal	1	0.018		0.551	1	0.004		0.110
Body	0.42 (0.22–0.80)				0.48 (0.29–0.78)			
Distal	0.62 (0.39–1.00)				0.61 (0.42–0.89)			
T stage								
1	1	< 0.001		0.518	1	< 0.001	1	< 0.001
2	5.79 (1.06–31.59)				1.35 (0.55–3.32)		0.76 (0.29–1.94)	
3	14.89 (3.58–61.83)				2.85 (1.60–5.08)		1.87 (1.03–3.41)	
4	22.28 (5.37–92.40)				4.78 (2.71–8.45)		3.02 (1.66–5.49)	
N stage								
0	1	< 0.001		0.369	1	< 0.001		0.614
1	5.15 (2.69–9.89)				1.97 (1.24–3.14)			
2	5.02 (2.51–10.03)				2.55 (1.59–4.10)			
3	8.67 (4.41–17.04)				3.63 (2.26–5.84)			
TNM stage								
I	1	< 0.001	1	< 0.001	1	< 0.001		0.567
II	12.51 (2.97–52.72)		9.08 (2.13–38.67)		2.43 (1.38–4.26)			
III	28.80 (7.00–118.45)		15.83 (3.73–67.11)		5.05 (2.96–8.61)			
Differentiation								
Well/moderate	1	0.001		0.352	1	0.006		0.577
Poor	2.20 (1.41–3.45)				1.62 (1.15–2.28)			
Vascular invasion								
No	1	< 0.001	1	0.024	1	< 0.001	1	< 0.001
Yes	2.89 (1.86–4.50)		1.70 (1.07–2.70)		2.43 (1.72–3.43)		2.04 (1.39–2.97)	
Lymph node ratio								
0	1	<0.001		0.448	1	< 0.001		0.444
0.01–0.24	4.54 (2.39–8.62)				1.88 (1.20–2.93)			
0.25–0.49	6.84 (3.50–13.37)				3.13 (1.96–4.99)			
≥ 0.50	8.83 (4.29–18.17)				3.75 (2.24–6.30)			
R status								
0	1	< 0.001	1	0.002	1	< 0.001	1	< 0.001
1	4.21 (2.73–6.49)		2.50 (1.51–4.14)		2.78 (1.89–4.10)		2.18 (1.40–3.40)	
Platelet count								
Low	1	0.038		0.505				
High	1.87 (1.03–3.39)							
Modified Glasgow Prognostic Score								
Low	1	0.001	1	0.011	1	< 0.001	1	< 0.001
Intermediate	1.98 (1.08–3.63)		1.76 (0.96–3.25)		1.91 (1.17–3.08)		2.06 (1.25–3.39)	
High	2.86 (1.56–5.24)		2.51 (1.35–4.65)		2.79 (1.72–4.54)		2.75 (1.65–4.59)	

### Relationships Between Clinicopathological Factors and Overall Survival

The relationship between overall survival and all studied factors can be found in supplementary Table 2. On univariable analysis, proximal location, advancing pT stage, advancing pN stage, advancing pTNM stage, poor differentiation, vascular invasion, advancing lymph node ratio, R1 status, raised CRP, and advancing mGPS (Fig. 1) were all associated with poor overall survival (*p* < 0.05) (Table 3). On multivariable analysis, only pT stage (when compared with T1, T3 HR 1.87 (1.03–3.41) and pT4 HR 3.02 (1.66–5.49) *p* < 0.001), vascular invasion (HR 2.04 (1.39–2.97) *p* < 0.001), R1 status (HR 2.18 (1.40–3.40) *p* < 0.001), and advancing mGPS (when compared with mGPS = 0, mGPS = 1 (HR 2.06 (1.25–3.39) and mGPS = 2 (HR 2.75 (1.65–4.59), *p* < 0.001) were independently associated with poor overall survival (Table 3). The number of events per variable was 12.1.

### The Relationship Between Clinicopathological Factors and the mGPS

The relationship between mGPS and clinicopathological factors is shown in Table 4. The mGPS was associated with advancing pT stage, advancing pTNM stage, poor differentiation, lower proportion of neoadjuvant treatments, raised white cell count, raised neutrophil count, raised platelet count, raised NLR, raised NPS, and a raised PLR (all *p* < 0.05) (Table 4).

**Table 4:** The relationship between the modified Glasgow Prognostic Score and clinicopathological factors in patients undergoing potentially curative resection for gastric cancer

Clinicopathological factors	mGPS = 0	mGPS = 1	mGPS = 2	*p* value
Age (years)				
< 65	78 (33.8%)	11 (35.5%)	10 (34.5%)	0.885
65–75	94 (40.7%)	13 (41.9%)	14 (48.3%)	
> 75 years	59 (25.5%)	7 (22.6%)	5 (17.2%)	
Sex				
Female	78 (33.8%)	9 (29.0%)	10 (34.5%)	0.863
Male	153 (66.2%)	22 (71.0%)	19 (65.5%)	
Tumor site				
Proximal	76 (32.9%)	8 (25.8%)	11 (37.9%)	0.697
Body	54 (23.4%)	7 (22.6%)	4 (13.8%)	
Distal	101 (43.7%)	16 (51.6%)	14 (48.3%)	
T stage				
1	63 (27.3%)	3 (9.7%)	1 (3.4%)	0.001
2	20 (8.7%)	4 (12.9%)	3 (10.3%)	
3	81 (35.1%)	14 (45.2%)	10 (34.5%)	
4	67 (29.0%)	10 (32.3%)	15 (51.7%)	
N stage				
0	107 (46.3%)	12 (38.7%)	12 (41.4%)	0.471
1	48 (20.8%)	8 (25.8%)	7 (24.3%)	
2	45 (19.5%)	4 (12.9%)	3 (10.3%)	
3	31 (13.4%)	7 (22.6%)	7 (24.1)	
Tumor stage				
I	73 (31.6%)	4 (12.9%)	2 (6.9%)	0.013
II	66 (28.6%)	14 (45.2%)	12 (41.4%)	
III	92 (39.8%)	13 (41.9%)	15 (51.7%)	
Differentiation				
Well/moderate	127 (55.0%)	12 (38.7%)	11 (37.9%)	0.030
Poor	104 (45.0%)	19 (61.3%)	18 (62.1%)	
Vascular invasion				
No	135 (58.4%)	21 (67.7%)	12 (41.4%)	0.105
Yes	96 (41.6%)	10 (32.3%)	17 (58.6%)	
Lymph node ratio				
0	107 (46.3%)	12 (38.7%)	12 (41.4%)	0.196
0.01–0.24	57 (24.7%)	10 (32.3%)	8 (27.6%)	
0.25–0.49	49 (21.2%)	2 (6.5%)	3 (10.3%)	
≥ 50	18 (7.8%)	7 (22.6%)	6 (20.7%)	
Lymph node sample				
≥ 15	107 (46.3%)	11 (35.5%)	16 (55.2%)	0.306
< 15	124 (53.7%)	20 (64.5%)	13 (44.8%)	
R status				
0	199 (86.1%)	26 (83.9%)	21 (72.4%)	0.068
1	32 (13.9%)	5 (16.1%)	8 (27.6%)	
Adjuvant chemotherapy				
No	117 (76.6%)	27 (87.1%)	26 (89.7%)	0.052
Yes	54 (23.4%)	4 (12.9%)	3 (10.3%)	
Neoadjuvant therapy				
No	176 (76.2%)	27 (87.1%)	28 (96.6%)	0.020
Yes	55 (23.8%)	4 (12.9%)	1 (3.4%)	
White cell count				
Low	172 (74.5%)	23 (74.2%)	10 (34.5%)	0.002
Normal	41 (17.7%)	7 (22.6%)	15 (51.7%)	
High	18 (7.8%)	1 (3.2%)	4 (13.8%)	
Neutrophil count				
Low	216 (92.6%)	31 (100.0%)	19 (65.5%)	< 0.001
High	17 (7.4%)	0 (0.0%)	10 (34.5%)	
Lymphocyte count				
Low	17 (7.4%)	5 (16.1%)	5 (17.2%)	0.160
Normal	201 (87.0%)	26 (83.9%)	23 (79.3%)	
High	13 (5.6%)	0 (0.0%)	1 (3.4%)	
Platelet count				
Low	210 (90.9%)	28 (90.3%)	22 (75.9%)	0.046
High	21 (9.1%)	3 (9.7%)	7 (24.1%)	
Neutrophil-lymphocyte ratio				
Low	219 (94.8%)	27 (87.1%)	15 (51.7%)	< 0.001
High	12 (5.2%)	4 (12.9%)	14 (48.3%)	
Neutrophil-platelet score				
Low	197 (85.3%)	28 (90.3%)	14 (48.3%)	< 0.001
Intermediate	30 (13.0%)	3 (9.3%)	12 (41.4%)	
High	4 (1.7%)	0 (0.0%)	3 (10.0%)	
Platelet-lymphocyte ratio				
Low	128 (55.4%)	7 (22.6%)	8 (27.6%)	< 0.001
High	103 (44.6%)	24 (77.4%)	21 (72.4%)	

## Discussion

The principal finding of this study was that the modified Glasgow prognostic score was the only inflammatory-based prognostic biomarker in a cohort of UK patients with gastric cancer, supporting the primary hypothesis. No fewer than one in five patients had raised markers of SIR, and some 1 in 10 the maximum SIR score. Patients with mGPS scores of zero experienced median DFS and OS, on average 15 and 16 months, respectively, better than patients with mGPS of two. Compared with a mGPS of zero, patients with a mGPS of two were nearly 3 times more likely to have nonresectable disease at laparotomy, 78% more likely to have T4 disease, 30% more likely to have TNM stage III disease, and be associated with both poor disease-free and overall survival. Similarly, patients with mGPS scores of zero experienced a 5-year DFS and OS of 80 and 52%, eight and twofold better than patients with mGPS scores of two. Sensitivity and specificity for patients with a mGPS of zero and two were 26 and 93% for DFS, and 2% and 94% for OS.

The modified Glasgow prognostic score has been associated with poor survival in a spectrum of anatomical cancer sites including colorectal,^12^ lung,^7^ breast,^15^ prostate,^16^ and gastric cancer.^17^ Based on the mGPS as a marker of inflammation, 20% of patients undergoing resection for gastric cancer had evidence of systemic inflammation on preoperative blood analysis. This compares with 36.5% of patients in colorectal cancer.^18^ Park et al. reported that 63.8, 20.7, and 15.5% of colorectal cancer patients had mGPS scores of zero, one, and two respectively, with associated a respective 5-year survival of 70% (mGPS zero), 60% (mGPS one), and 46% (mGPS two). The observation of a stepwise 10–15% difference in a 5-year survival relative to each mGPS subgroup was mirrored in this study, although the overall survival of mGPS zero patients was 50%, arguably reflecting the inherent aggressive nature of gastric cancer. Although the relationship between mGPS and gastric cancer survival has been reported before, this is the first report to highlight the superior prognostic value of the mGPS when compared with all markers of inflammation readily available to clinicians. The conceptual role of the host and cancer-related factors in predicting survival is not new. Roxburgh et al. 11 and Dutta et al. 9 examined host and cancer factors related to survival in colorectal and gastric cancer, respectively. Both reported that systemic inflammation was an important predictor of survival independent of stage. This study builds on this work by including all of the clinically available inflammation and pathological factors in a more comprehensive multivariable model.

There is a now an established evidence base detailing the poor prognosis associated with the presence of a preoperative SIR. The findings of this study support the role of inflammation in tumor progression and metastasis. The mechanism of how the SIR exerts an adverse influence on survival is unclear, but is likely part of a complex biosystem involving cellular signaling and genetic aberrations, resulting in tumor growth, tissue remodeling, angiogenesis, and dysregulation of the innate/adaptive immune responses. It is possible that the poor prognoses associated with SIR is not based on local disease progression alone, and the finding that SIR was also associated with the development of metastases, independent of TNM stage, supports an interaction between tumor and host. Understanding the underlying physiological mechanisms would raise the potential of therapeutic targets and further work is needed in this regard.

It is now well-established that the SIR offers valuable prognostic information. Yet, if the SIR and specifically the mGPS is to be incorporated into a modified TNM staging system, then it also needs to offer predictive value. Biomarker inclusion into the management of breast cancer was driven by the identification of adjuvant therapies for higher-risk patients. Apart from Herceptin treatment for advanced gastric cancer, the principal adjuvant treatment is cytotoxic chemotherapy. If the mGPS is to identify patients that may benefit from chemotherapy, then evidence of potential response is required. Unfortunately, evidence from the arena of colorectal cancer suggests that patients with a SIR experienced poorer outcomes when undergoing neoadjuvant,^19^ or adjuvant chemotherapy 20 when compared with controls. Moreover, based on histological assessment, the mGPS in rectal cancer patients was associated with a greater and significant likelihood of nonresponse to neoadjuvant chemotherapy.^21^ Although, the mGPS has been associated with poorer survival in patients undergoing chemotherapy for gastric cancer,^22^ its role in predicting response to neoadjuvant therapy is unknown. Given the associations between systemic inflammation and poor response to chemotherapy, it is unlikely that these patients will derive benefit from adjuvant chemotherapy following potentially curative resection suggesting that alternative therapeutic modalities are needed. The phase III, double-blinded, placebo-controlled randomized trial assessing the effects of aspirin on disease recurrence and survival after primary therapy in common nonmetastatic solid tumors (Add-Aspirin) commenced recruiting in October 2015.^23^ Although this study should offer valuable information on whether aspirin might benefit and be offered to all patients undergoing potentially curative gastrectomy, it is possible that only patients with a SIR will likely respond. Similar to other adjuvant treatments such as Herceptin and Cetuximab, patient selection will be crucial and the mGPS has the potential to be a valuable predictive biomarker in this setting.

There are a number of inherent limitations and potential criticisms of this study. Cohort sample size was modest and the study was powered based on a 15% difference in survival, subanalysis related to tumor stage was therefore not possible. Data related to patients’ race, body mass indices, and detailed comorbidity was not collected in the prospective database, and was therefore not available for analysis as confounding factors, but is worthy of further study. Validating these results in an appropriately powered cohort for subanalysis related to stage II and III disease may help integrate the mGPS into a modified TNM staging system. The patient cohort studied is a selected group (most had undergone a potentially curative R0 gastrectomy) and were certainly not representative of all patients with gastric cancer; indeed, only approximately one third of the patients in South Wales undergo potentially curative resection.^24^ In contrast, the study has several strengths, benefiting from robust follow-up data with accurate causes and dates of death obtained from the office of national statistics; over 75% were followed up for at least 5 years or death. A NHS laboratory using standardized techniques performed the serum measurements, and therefore reproducing these results is not anticipated to be problematic. Moreover, the patients were recruited from a consecutive series of patients diagnosed with gastric cancer, from a single UK geographical region, and all had been treated by the same multidisciplinary team and group of specialist surgeons, using a standardized staging algorithm and operative technique, with extensive audited and published quality control.

In conclusion, the mGPS was the only inflammatory-based prognostic biomarker to be associated with poorer OS and DFS in patients undergoing potentially curative resection for gastric cancer, and was independent of pathological stage. In light of emerging evidence that SIR predicts poor survival and nonresponse in patients undergoing perioperative chemotherapy, other therapeutic modalities and options are desirable. These findings suggest that the SIR may offer an original and novel therapeutic target for patients more susceptible to cancer recurrence, and the modified Glasgow Prognostic Score may represent the most promising biomarker available for patient tailored holistic antiinflammatory treatment.

## Electronic supplementary material

ESM 1 (DOCX 93 kb)
