# Supplementary material for: Prognostic Significance of Serum Inflammatory Markers in Gastric Cancer
Source: J Gastrointest Surg. 2017 Oct 4;22(4):595–605. doi: 10.1007/s11605-017-3597-5 (PMC5869874; doi:10.1007/s11605-017-3597-5)
Supplement: Supplementary file 1 — (DOCX 93 kb) [file 11605_2017_3597_MOESM1_ESM.docx]

| Clinicopathological factors | Resection  n=291 | Non-resection  n=41 | p-value |
| --- | --- | --- | --- |
| Age (years)  <65  65 – 75  > 75 years | 99 (34.0%)  121 (41.6%)  71 (24.4%) | 12 (29.3%)  20 (48.8%)  9 (22.0) | 0.680 |
| Sex  Female  Male | 97 (33.3%)  194 (66.7%) | 4 (9.8%)  37 (90.2%) | 0.002 |
| Neoadjuvant therapy  No  Yes | 246 (84.5%)  45 (15.5%) | 40 (97.6%)  1 (2.4%) | 0.024 |
| White Cell count  Low  Normal  High | 205 (70.4%)  63 (21.6%)  23 (7.9%) | 30 (73.2%)  8 (19.5%)  23 (7.9%) | 0.937 |
| Neutrophil Count  Normal  High | 264 (90.7%)  27 (9.3%) | 37 (90.2%)  4 (9.8%) | 0.247 |
| Lymphocyte Count  Low  Normal  High | 27 (9.3%)  250 (85.9%)  14 (4.8%) | 4 (9.8%)  36 (87.8%)  1 (2.4%) | 0.644 |
| Platelet Count  Normal  High | 260 (89.3%)  31 (10.7%) | 35 (85.4%)  6 (14.6%) | 0.448 |
| C-Reactive Protein  Normal  High | 231 (79.4%)  60 (20.6%) | 24 (58.5%)  17 (41.5%) | 0.003 |
| Albumin  Normal  Low | 219 (75.3%)  72 (24.7%) | 24 (58.5%)  17 (41.5%) | 0.024 |
| Neutrophil-Lymphocyte Ratio  Low  High | 261 (89.7%)  30 (10.3%) | 38 (92.7%)  3 (7.3%) | 0.549 |
| Neutrophil-Platelet Score  Low  Intermediate  High | 239 (82.1%)  45 (15.5%)  7 (2.4%) | 33 (80.5%)  6 (14.6%)  2 (4.9%) | 0.658 |
| Platelet-Lymphocyte Ratio  Low  High | 143 (49.1%)  148 (50.9%) | 15 (36.6%)  26 (63.4%) | 0.132 |
| Modified Glasgow Prognostic Score  Low  Intermediate  High | 231 (79.4%)  31 (10.7%)  29 (10.0%) | 24 (58.5%)  6 (14.6%)  11 (26.8%) | 0.001 |

Supplementary table 1

Supplementary Table 2

|  | Disease free survival |  | Overall survival |  |
| --- | --- | --- | --- | --- |
|  | Hazard ratio (95% CI) | p-value | Hazard ratio (95% CI) | p-value |
| Age  <65  65 – 75  >75 | 1  0.83 (0.52 - 1.34)  0.54 (0.28 - 1.03) | 0.173 | 1  0.99 (0.67 - 1.45)  0.99 (0.63 - 1.56) | 0.998 |
| Sex  Female  Male | 1  0.61 (0.39 - 0.95) | 0.027 | 1  0.77 (0.54 - 1.09) | 0.137 |
| Tumour site  Proximal  Body  Distal | 1  0.42 (0.22 - 0.80)  0.62 (0.39 - 1.00) | 0.018 | 1  0.48 (0.29 - 0.78)  0.61 (0.42 - 0.89) | 0.004 |
| T stage  1  2  3  4 | 1  5.79 (1.06 - 31.59)  14.89 (3.58 - 61.83)  22.28 (5.37 - 92.40) | <0.001 | 1  1.35 (0.55 - 3.32)  2.85 (1.60 - 5.08)  4.78 (2.71 - 8.45) | <0.001 |
| N stage  0  1  2  3 | 1  5.15 (2.69 - 9.89)  5.02 (2.51 - 10.03)  8.67 (4.41 – 17.04) | <0.001 | 1  1.97 (1.24 - 3.14)  2.55 (1.59 - 4.10)  3.63 (2.26 - 5.84) | <0.001 |
| TNM stage  I  II  III | 1  12.51 (2.97 - 52.72)  28.80 (7.00 - 118.45) | <0.001 | 1  2.43 (1.38 - 4.26)  5.05 (2.96 – 8.61) | <0.001 |
| Differentiation  Well/Moderate  Poor | 1  2.20 (1.41 - 3.45) | 0.001 | 1  1.62 (1.15 - 2.28) | 0.006 |
| Vascular invasion  No  Yes | 1  2.89 (1.86 - 4.50) | <0.001 | 1  2.43 (1.72 - 3.43) | <0.001 |
| Lymph Node Ratio  0  0.01 – 0.24  0.25 – 0.49  ≥ 0.50 | 1  4.54 (2.39 - 8.62)  6.84 (3.50 - 13.37)  8.83 (4.29 - 18.17) | <0.001 | 1  1.88 (1.20 - 2.93)  3.13 (1.96 - 4.99)  3.75 (2.24 - 6.30) | <0.001 |
| Lymph node sample  ≥15  <15 | 1  1.20 (0.77 - 1.88) | 0.411 | 1  0.87 (0.62 - 1.22) | 0.408 |
| R status  0  1 | 1  4.21 (2.73 - 6.49) | <0.001 | 1  2.78 (1.89 - 4.10) | <0.001 |
| Adjuvant chemotherapy  No  Yes | 1  1.30 (0.77 - 2.17) | 0.326 | 1  1.29 (0.82 - 2.01) | 0.727 |
| Neoadjuvant therapy  No  Yes | 1  1.07 (0.58 - 1.97) | 0.837 | 1  0.98 (0.59 - 1.63) | 0.930 |
| White Cell count  Low  Normal  High | 1  1.15 (0.69 - 1.92)  0.85 (0.34 – 1.11) | 0.778 | 1  1.08 (0.72 - 1.62)  1.15 (0.60 - 2.22) | 0.871 |
| Neutrophil Count  Low  High | 1  0.76 (0.33 - 1.75) | 0.524 | 1  0.77 (0.40 - 1.46) | 0.423 |
| Lymphocyte Count  Low  Normal  High | 1  0.93 (0.45 - 1.94)  1.83 (0.63 - 5.27) | 0.287 | 1  0.80 (0.47 - 1.37)  0.92 (0.36 - 2.36) | 0.694 |
| Platelet Count  Low  High | 1  1.87 (1.03 - 3.39) | 0.038 | 1  1.47 (0.89 - 2.46) | 0.136 |
| C-Reactive Protein  Normal  High | 1  2.34 (1.47 - 3.73) | <0.001 | 1  2.26 (1.56 - 3.28) | <0.001 |
| Albumin  Normal  Low | 1  1.36 (0.83 - 2.22) | 0.224 | 1  1.41 (0.95 - 2.09) | 0.084 |
| Derivative markers |  |  |  |  |
| Neutrophil-Lymphocyte Ratio  Low  High | 1  1.43 (0.78 - 2.65) | 0.249 | 1  1.01 (0.58 - 1.76) | 0.975 |
| Neutrophil-Platelet Score  Low  Intermediate  High | 1  1.70 (1.01 - 2.87)  0.58 (0.81 - 4.19) | 0.115 | 1  1.54 (1.00 - 2.36)  0.30 (0.04 - 2.15) | 0.065 |
| Platelet-Lymphocyte Ratio  Low  High | 1  1.26 (0.81 - 1.95) | 0.302 | 1  1.28 (0.91 - 1.80) | 0.164 |
| Modified Glasgow Prognostic Score  Low  Intermediate  High | 1  1.98 (1.08 - 3.63)  2.86 (1.56 - 5.24) | 0.001 | 1  1.91 (1.17 - 3.08)  2.79 (1.72 - 4.54) | <0.001 |
